# CRISPR/Cas9-mediated genome editing via postnatal administration of AAV vector cures haemophilia B mice

**DOI:** 10.1038/s41598-017-04625-5

**Published:** 2017-06-23

**Authors:** Tsukasa Ohmori, Yasumitsu Nagao, Hiroaki Mizukami, Asuka Sakata, Shin-ichi Muramatsu, Keiya Ozawa, Shin-ichi Tominaga, Yutaka Hanazono, Satoshi Nishimura, Osamu Nureki, Yoichi Sakata

**Affiliations:** 1 Department of Biochemistry, Jichi Medical University School of Medicine, Tochigi, 329-0498 Japan; 2 Center for Experimental Medicine, Jichi Medical University, Tochigi, 329-0498 Japan; 3 Division of Genetic Therapeutics, Center for Molecular Medicine, Jichi Medical University, Tochigi, 329-0498 Japan; 4 Division of Cell and Molecular Medicine, Center for Molecular Medicine, Jichi Medical University, Tochigi, 329-0498 Japan; 5 Department of Neurology, Jichi Medical University School of Medicine, Tochigi, 329-0498 Japan; 6 The Institute of Medical Science, The University of Tokyo, Tokyo, 108-0071 Japan; 7 Division of Regenerative Medicine, Center for Molecular Medicine, Jichi Medical University, Tochigi, 329-0498 Japan; 8 Department of Cardiovascular Medicine, The University of Tokyo, Tokyo, 113-8655 Japan; 9 Translational Systems Biology and Medicine Initiative, The University of Tokyo, Tokyo, 113-8655 Japan; 10 Department of Biological Sciences, Graduate School of Science, The University of Tokyo, Tokyo, 113-0032 Japan

## Abstract

Haemophilia B, a congenital haemorrhagic disease caused by mutations in coagulation factor IX gene (*F9*), is considered an appropriate target for genome editing technology. Here, we describe treatment strategies for haemophilia B mice using the clustered regularly interspaced short palindromic repeat (CRISPR)/Cas9 system. Administration of adeno-associated virus (AAV) 8 vector harbouring *Staphylococcus aureus* Cas9 (SaCas9) and single guide RNA (sgRNA) to wild-type adult mice induced a double-strand break (DSB) at the target site of *F9* in hepatocytes, sufficiently developing haemophilia B. Mutation-specific gene editing by simultaneous induction of homology-directed repair (HDR) sufficiently increased FIX levels to correct the disease phenotype. Insertion of *F9* cDNA into the intron more efficiently restored haemostasis via both processes of non-homologous end-joining (NHEJ) and HDR following DSB. Notably, these therapies also cured neonate mice with haemophilia, which cannot be achieved with conventional gene therapy with AAV vector. Ongoing haemophilia therapy targeting the antithrombin gene with antisense oligonucleotide could be replaced by SaCas9/sgRNA-expressing AAV8 vector. Our results suggest that CRISPR/Cas9-mediated genome editing using an AAV8 vector provides a flexible approach to induce DSB at target genes in hepatocytes and could be a good strategy for haemophilia gene therapy.

## Introduction

Hemophilia is an X-linked congenital hemorrhagic disease affecting 1 in 5000–10 000 males. The disease is caused by mutations in coagulation factor VIII (*FVIII*) or IX (*FIX*) genes (*F8* and *F9* genes, respectively). Defects in these coagulation factors trigger severe bleeding episodes (e.g., joint bleeding, muscle bleeding, purpura, and intracranial hemorrhage). Although several studies have reported on an extremely shortened life expectancy for hemophilia patients (range, 16–23 years), from the latter half of the 20th century the prognosis for such patients has improved markedly following the introduction of better quality coagulation factor concentrates^1^. However, because of the extremely short half-life of these coagulation factors, patients need prophylactic intravenous administration of the treatment as frequently as 1–3 times per week from early childhood^2^. Permanent repair of the gene responsible for hemophilia is a worthwhile goal for medical science.

As a system, CRISPR/Cas9 shows great potential to correct disease-causing mutations^3, 4^. Cas9 protein interacts with specific sites in the genome adjacent to a protospacer adjacent motif (PAM) in the presence of sgRNA and induces double strand breaks (DSB)^5, 6^. Induction of a DSB by the CRISPR/Cas9 system can promote two DNA repair pathways: homology-directed repair (HDR) and non-homologous end-joining (NHEJ)^6^. The CRISPR/Cas9 system was able to correct a disease-specific mutation in the germ line by HDR in a mouse model of Duchenne muscular dystrophy and hearing loss^7, 8^. However, germ line genome editing cannot be applied to humans at present because of ethical and safety concerns^9^. Therefore, effective delivery of the genome editing components into target somatic cells to treat genetic diseases is a desirable alternative.

One difficulty in applying the CRISPR/Cas9 system to somatic cells is the lower frequencies of HDR. Consequently, NHEJ is the main mechanism used to repair DSB^10^, and HDR rarely occurs in post-mitotic adult tissues, such as skeletal muscle and the liver^3^. Deletion of an abnormal mutation is easier than correction of a disease-specific mutation. Recently, three groups simultaneously reported phenotypic correction using CRISPR/Cas9 in a mouse model of Duchenne muscular dystrophy after birth^11–13^. They induced DSB on both sides of an abnormal exon by two AAV vectors to promote permanent exon skipping. However, the method cannot be applied to haemophilia treatment because even one amino acid substitution may affect the activities of coagulation factors^14^. Therefore, an alternative strategy to efficiently express normal gene products or to inhibit a negative regulator of a coagulation system should be considered for haemophilia treatment. In this study, we effectively delivered genome editing components including Cas9 and sgRNA into hepatocytes using a single AAV8 vector, and were able to restore hemostasis in a mouse model of hemophilia B using three different strategies.

## Results

### Generation of FIX-deficient mice

We first generated haemophilia B mice with deletion of *F9* to create a treatment model for genome editing (Extended Fig. 1). *Streptococcus pyogenes* Cas9 mRNA and a sgRNA specific to exon 8 of mouse *F9* were injected into fertilized embryos (Extended Fig. 1A and B, and Extended Table 1). Newborn mice carrying the mutation showed reduced plasma coagulation factor IX (FIX) activity (FIX:C) (Extended Fig. 1C and D). DNA sequencing of a strain of male F2 mice, in which no FIX:C was detected, revealed a 12-base deletion in the sgRNA sequence (Extended Fig. 1E).

### Disruption of F9 in liver using an AAV vector *in vivo*

**Figure 1:**
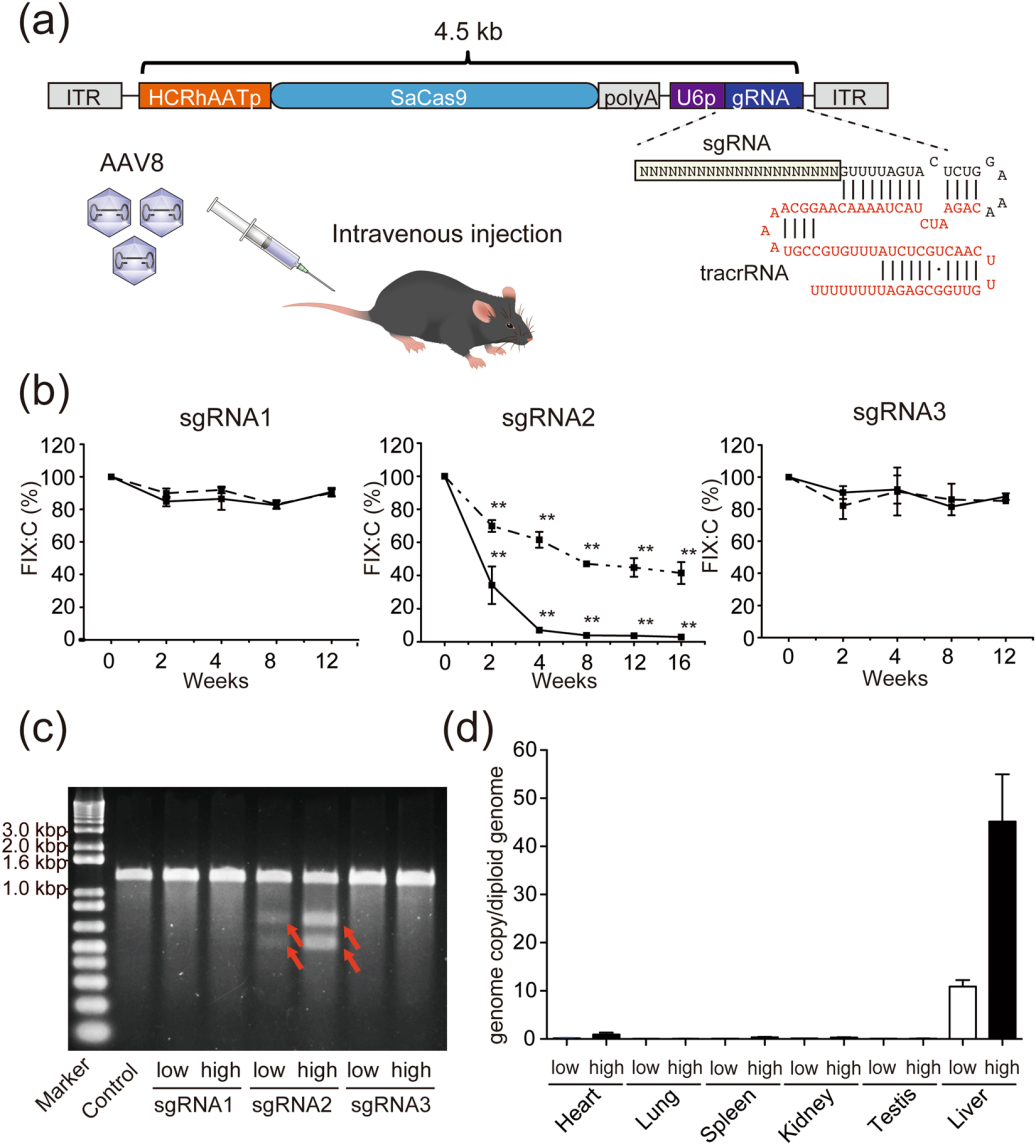
AAV vector-mediated generation of haemophilia B in adult wild-type mice. (**a**) Schematic diagram of a single AAV vector expressing SaCas9 and sgRNA. HCRhAATp, an enhancer element of the hepatic control region of the Apo E/C1 gene and the human anti-trypsin promoter. (**b**) AAV vector expressing SaCas9 and each sgRNA targeting *F9* was intravenously injected into 7-week-old C57BL/6 J male mice, and plasma levels of FIX:C were measured at indicated times. Solid and dashed lines represent high (1 × 10^12^ vector genome/body) and low dose treatments (3 × 10^11^ vector genome/body), respectively. Values are mean ± SEM (n = 3–6). **P < 0.01, compared with pretreatment (*post hoc* Bonferroni test). (**c**) Cas9-mediated cleavage of *F9* in the liver was assessed using the Surveyor^®^ nuclease assay. Control was liver DNA from non-treated C57BL/6 J mice. Red arrows represent a mutation. (**d**) Distribution of the AAV genome in each tissue at 8 weeks after the injection. Values are mean ± SEM (n = 6).

FIX is a vitamin K-dependent coagulation factor produced in hepatocytes. CRISPR/Cas9-mediated somatic correction of *F9* by hydrodynamic injection of naked DNA has been reported^15^. However, hydrodynamic administration is not a realistic option for human therapy. In addition, adenoviral expression of Cas9 does not show any therapeutic effect because of severe hepatic toxicity^15^. To solve these problems, we used an AAV vector to deliver genome editing tools to the liver *in vivo*. We prepared an AAV8 vector that expresses SaCas9 in a hepatocyte-specific manner under control of a chimeric promoter (HCRhAAT; an enhancer element of the hepatic control region of the Apo E/C1 gene and the human anti-trypsin promoter)^16^ and simultaneously expresses a sgRNA specific to exon 8 of mouse *F9* under U6 promoter (Fig. 1a and Extended Table 1). HCRhAAT promoter showed higher transgene expression in the liver compared with human thyroxine-binding globulin promoter (Extended Fig. 2), which was reportedly used to express SaCas9^17^. We intravenously injected the AAV8 vector that expressed sgRNA targeting exon 8 of *F9* into wild-type mice. FIX:C levels decreased to 2–5% after administration of high-dose AAV8 vector expressing sgRNA2 (1 × 10^12^/body), suggesting SaCas9 efficiently induced DSB in *F9* of hepatocytes *in vivo* (Fig. 1b). DNA mutation following DSB in liver tissue was confirmed using the Surveyor^®^ nuclease assay (Fig. 1c), and exhibited deletions or replacement near the PAM sequence using next-generation sequencing (Extended Table 2). Only half of the *F9* allele seemed not to be disrupted using the Surveyor^®^ assay (Fig. 1c). We think that this is because we mixed the analysed sample with the wild-type PCR product. Immunohistochemical staining showed that SaCas9 was expressed in nearly all hepatocytes, but not in endothelial cells (Extended Fig. 3). No histological abnormalities were detected with haematoxylin and eosin staining (Extended Fig. 3). The AAV genome was mainly detected in the liver (Fig. 1d), and no cleavage of the target genomic DNA was observed in any organ apart from the liver (Extended Fig. 4).

The most important drawback of using CRISPR/Cas9 in clinical use is unwanted mutations at off-target sites that resemble the on-target sequence^18, 19^. We used Cas-OFFinder (http://www.rgenome.net/cas-offinder/) to list potential off-target sites that differed from on-target sites by up to four nucleotides or by two nucleotides with a bulge of up to two bases. We extracted 28 candidate off-target sites (Extended Table 3) but did not detect any mutations using the Surveyor^®^ assay (Extended Fig. 5).

### Marginal increase in plasma FIX levels by HDR

**Figure 2:**
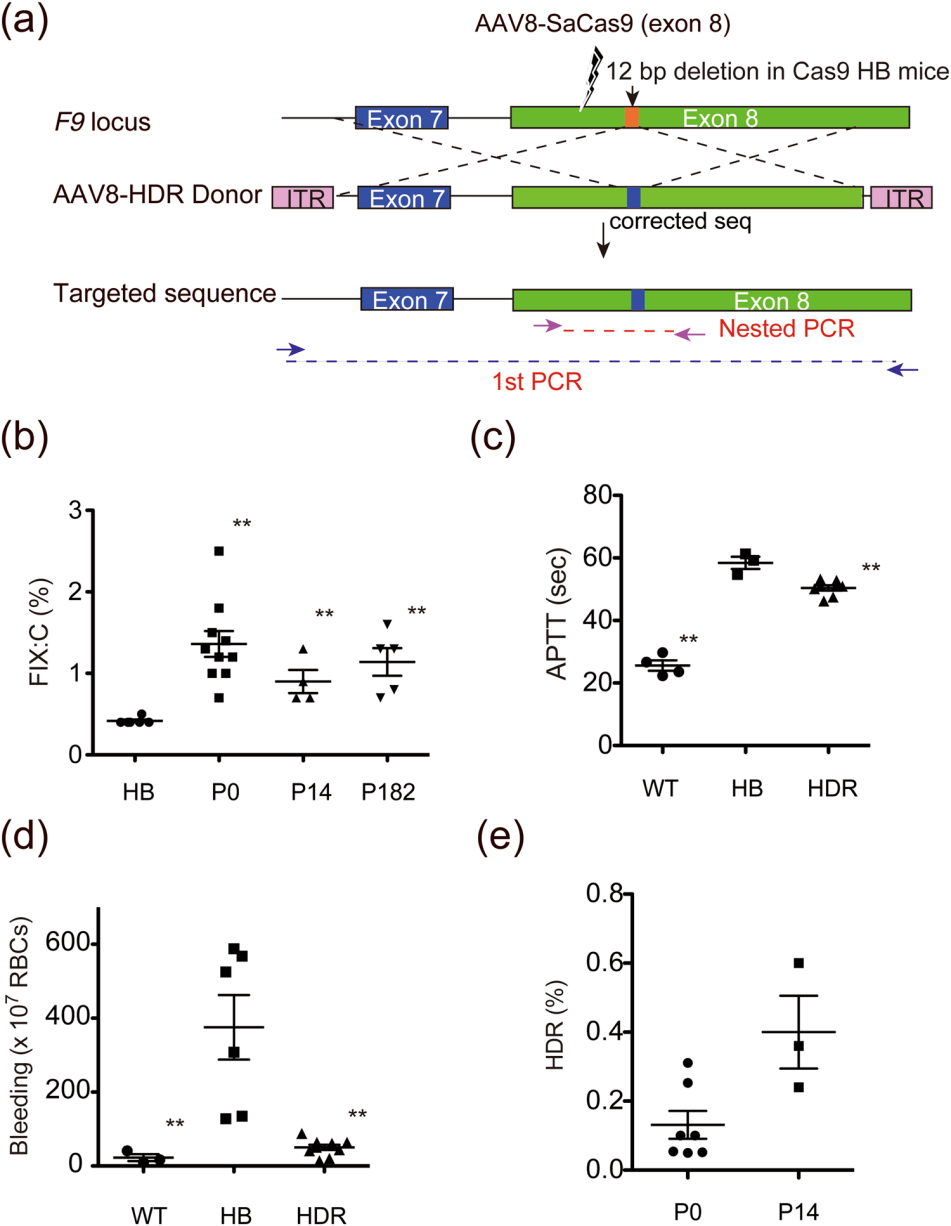
Phenotypic correction of haemophilia B mice by homologous directed repair (HDR). (**a**) Schematic representation of the targeting strategy. The AAV8 vector expressing SaCas9 and sgRNA targeting exon 8 of *F9* (AAV8-SaCas9 (exon 8)) induces a DSB. Simultaneous administration of the AAV8 vector containing homologous donor sequence (AAV8-HDR Donor) enabled correction of the target sequence by HDR. (**b**) AAV8-SaCas9 (exon 8) and AAV8-HDR Donor were injected into 0- (intraperitoneal injection, n = 10), 14- (intravenous injection, n = 4) or 26- to 45-week-old (182 days) (intravenous injection, n = 5) haemophilia B (HB) mice. Plasma FIX:C levels at 4–8 weeks following intravenous injections were measured. Vector dose (AAV8-SaCas9 (exon 8)/AAV8-HDR Donor): 6 × 10^10^ vg/2 × 10^11^ in 0-day-old; 2.4 × 10^11^ vg/6 × 10^11^ in 14-day-old; 1 × 10^12^ vg/3 × 10^12^ in 26- to 45-week-old mice. (**c**,**d**) Blood coagulation assessed by activated partial thromboplastin time (APTT) (**c**) and bleeding volume after tail clipping for 10 min (**d**) were measured in wild-type mice (WT), HB mice, and HB mice treated with HDR mechanism (HDR) (n = 3–8). Values are mean ± SEM. **P < 0.01, compared with HB mice (two-tailed Student’s *t*-test). (**e**) To analyse the frequencies of HDR in liver DNA, two-step PCR was performed. Liver DNA was obtained from HB mice treated at P0 (n = 7) or P14 (n = 3). PCR fragments were amplified not to contain the donor vector followed by nested PCR. The frequency of the sequence that underwent HDR in the nested PCR sample was quantified using next-generation sequencing. Values are mean ± SEM.

We further examined whether simultaneous delivery of a donor sequence to repair the mutation would increase FIX:C by HDR in haemophilia B mice created using zygote injection of CRISPR/Cas9 tools. A donor sequence with homology arms of 1 kb on both the 5′ and 3′ sides of the sgRNA sequence was introduced into the AAV8 vector (Fig. 2a). Silent mutations were incorporated in the sgRNA recognition site of the donor sequence so that homologous recombination of *F9* would not be re-cleaved by SaCas9. We detected a significant improvement in FIX:C, activated partial thromboplastin time, and bleeding phenotypes in haemophilia B mice after injection of the vector (Fig. 2b,c and d). Although HDR mainly occurs during cell proliferation (S/G2 phase of the cell cycle)^20^, the additional increase in FIX:C or HDR could not be obtained by neonatal injection compared with treatment at the adult phase (Fig. 2b and e).

### Insertion of an exon 2–8 cDNA into intron 1 of *F9*

**Figure 3:**
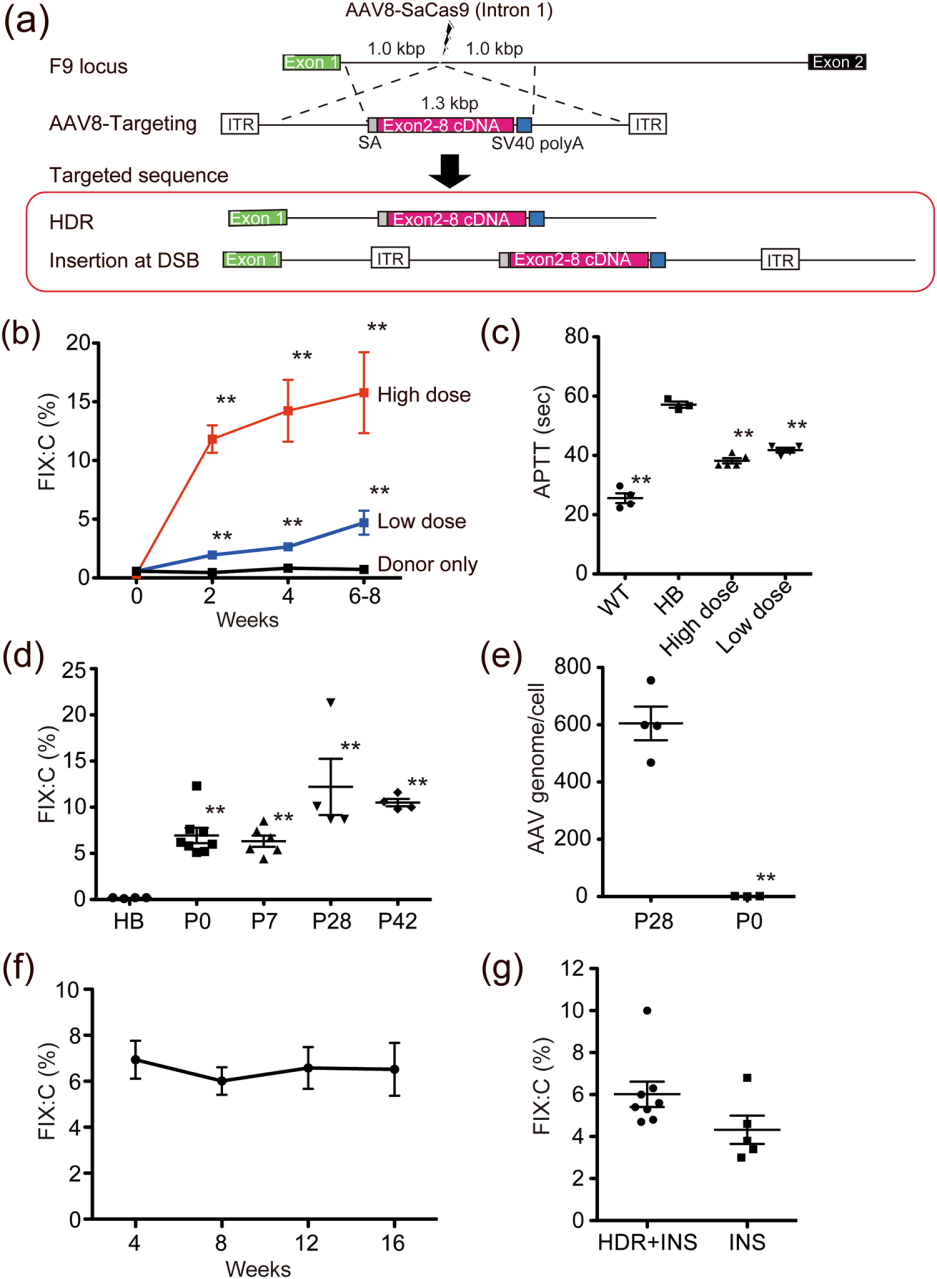
Efficient increase in plasma FIX:C in haemophilia B mice by both HDR and insertion at DSB. (**a**) Schematic representation of targeting strategy. AAV8-SaCas9-sgRNA3 for *F9* intron (AAV8-SaCas9 (intron 1)), the AAV8 vector to induce a DSB in intron 1; AAV8-Targeting, the gene correction AAV vector; SA, human *F9* intron 1 splice acceptor site; *F9* exon 2–8, codon-optimized cDNA (exon 2–8) of mouse *F9*. (**b**,**c**) Haemophilia B male (HB) mice (4–8 weeks old) were treated with intravenous injection of AAV8-SaCas9 (intron 1) and AAV8-Targeting. (**b**) Plasma levels of FIX:C were measured at indicated times after vector administration. Red and blue lines represent high and low dose treatments, respectively. Black line represents treatment with donor vector only. (**c**) Activated partial thromboplastin time (APTT) was measured in wild-type C57BL/6 mice (WT), HB mice, and HB mice treated with high or low dose vector. Vector dose (AAV8-SaCas9 (intron 1)/AAV8-Targeting): 1 × 10^12^ vector genome (vg)/3 × 10^12^ vg in high dose, 0.3 × 10^12^ vg/1 × 10^12^ vg in low dose. Values are mean ± SEM (n = 3–6). **P < 0.01, compared with HB mice (two-tailed Student’s *t*-test). (**d**) AAV8-SaCas9 (intron 1) and AAV8-Targeting were injected into 0- (P0: intraperitoneal injection, n = 6), 7- (P7: intravenous injection, n = 6), 28- (P28: intravenous injection, n = 4), or 42-day-old (P42: intravenous injection, n = 4) HB mice. Plasma FIX:C levels were measured at 4–8 weeks following vector injection. Vector dose (AAV8-SaCas9 (intron 1)/AAV8-Targeting): 6 × 10^10^ vg/2 × 10^11^ in 0-day-old; 1.4–2.2 × 10^11^ vg/4.1–6.6 × 10^11^ in 7-day-old; 1 × 10^12^ vg/3 × 10^12^ in 28- and 42-day-old mice. Values are mean ± SEM. **P < 0.01, compared with HB mice (two-tailed Student’s *t*-test). (**e**) AAV genome in the liver at 8–16 weeks after vector injection in HB mice treated at 28 days old (P28) or 0 days old (P0). Values are mean ± SEM (n = 3–4). **P < 0.01 (two-tailed Student’s *t*-test). (**f**) AAV8-SaCas9 (intron 1) and AAV8-Targeting were intraperitoneally injected into 0-day-old HB mice, and plasma levels of FIX:C were measured at indicated times after vector administration. Values are mean ± SEM (n = 3–6). (**g**) AAV8-SaCas9 (intron 1) and AAV8-Targeting (HDR + INS) or AAV8-Targeting without arm sequence (INS) were intraperitoneally injected into 0-day-old HB mice, and plasma levels of FIX:C were measured at 4–8 weeks following vector injection. Values are mean ± SEM (n = 5–8). *P* = 0.096 (two-tailed Student’s *t*-test).

To further increase treatment efficacy with the CRISPR/Cas9 system, we tried to insert a cDNA fragment into endogenous *F9* intron 1 by both HDR and NHEJ (Fig. 3a). We designed a chimeric DNA sequence (human *F9* splice acceptor site and codon-optimized mouse *F9* cDNA (exon 2–8)) containing homology arms of 1 kb on both sides, and inserted this into an AAV8 vector (AAV8-Targeting; Fig. 3a). We administrated AAV8-Targeting together with AAV8 vector to induce DSB at intron 1 in haemophilia B mice created using zygote injection of CRISPR/Cas9 tools (AAV8-SaCas9 (intron 1); Fig. 3a, Extended Fig. 6). FIX:C gradually increased to 11.7–39.6% at a higher vector dose, and activated partial thromboplastin time significantly improved in 4–8-week-old haemophilia B mice (Fig. 3b and c). To examine the mode of genome editing, we amplified the DNA sequence between the inserted cDNA and the outside of the 3′ arm (small arrow in Extended Fig. 7A) and found that the *F9* cDNA sequence was inserted into the DSB not only by HDR but also by NHEJ (Extended Fig. 7B). We also confirmed FIX mRNA expression consisting of exon 1 and codon-optimized exons 2–8 (Extended Fig. 7C). Similar treatment effects were observed at postnatal day 0 (P0), day 7 (P7), day 28 (P28), and day 42 (P42) (Fig. 3d). The AAV genome in the liver at 8–16 weeks after vector injection was significantly lower in mice treated at birth (P28: 604.5 ± 58.73; P0: 1.177 ± 0.3437; Fig. 3e). AAV vectors are predominantly maintained episomally; therefore, cell division will dilute the AAV genome resulting in loss of therapeutic expression^21^. However, the therapeutic effect of genome editing will persist permanently (Fig. 3f), suggesting that application of CRISPR/Cas9 overcomes the defect of current AAV vector-mediated gene therapy.

To investigate the importance of NHEJ-medicated insertion, we treated haemophilia B mice with AAV8-targeting not containing homologous arms at P0 (Fig. 3g). When compared with the vector with arms (HDR + INS), the increase in FIX:C after treatment seemed somewhat weak, but was statistically not significant (HDR + INS: 6.013 ± 0.60, INS: 4.320 ± 0.6741; *P* = 0.0963). These data indicated that the most straightforward way to treat genetic diseases with genome editing is to enhance the insertion of the target sequence at DSB.

### Phenotypic correction of hemophilia by disruption of *Serpinc1*

**Figure 4:**
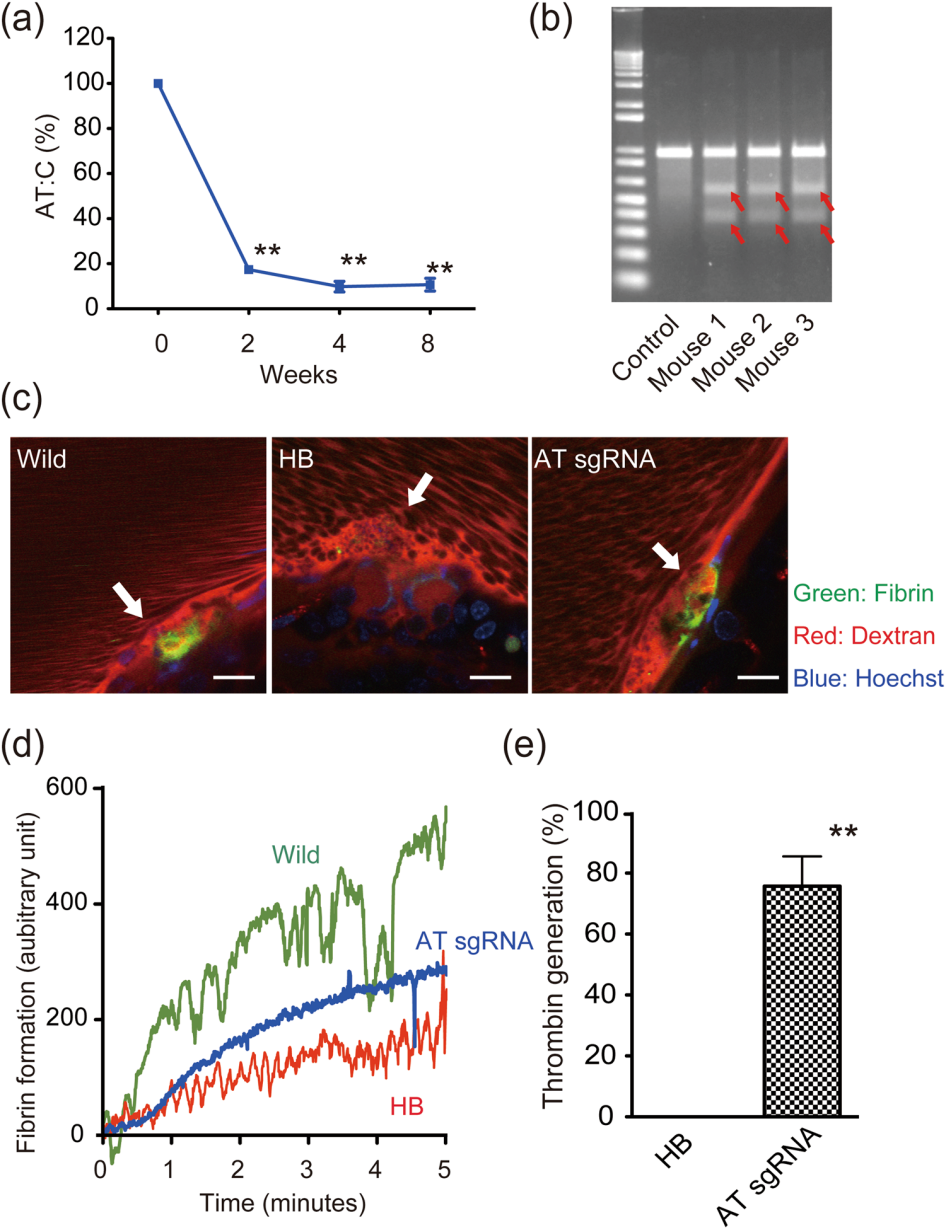
Disruption of the AT gene, *Serpinc1*, restores bleeding phenotypes of haemophilia B mice. AAV8 vector expressing SaCas9 and sgRNA targeting exon 8 of *Serpinc1* (sgRNA1 for *Serpinc1*) (AAV8-SaCas9 (AT)) was intravenously injected into 7–8-week-old haemophilia B (HB) male mice (1 × 10^12^ vector genome/body). (**a**) Plasma levels of antithrombin activity (AT:C) were measured at indicated times. Values are mean ± SEM (n = 8). **P < 0.01, compared with pretreatment (*post hoc* Bonferroni test). (**b**) Cas9-mediated cleavage of the genome in the liver was confirmed using the Surveyor^®^ nuclease assay. Red arrows represent a mutation. (**c**,**d**) Fibrin formation *in vivo* after endothelial disruption in wild-type C57BL/6 mice (WT), HB mice, and HB mice treated with AAV8-SaCas9 (AT) was observed by intravital confocal microscopy (Nikon A1RNP; Nikon, Tokyo, Japan) at ×400 magnification. Scale bars, 20 µm. (**c**) Representative images at 5 min after laser irradiation. Green signal indicates fibrin formation at the site of endothelial disruption (white arrow). (**d)** Signal intensities of fibrin formation were quantified using NIS-Elements AR 3.2 (Nikon), and are expressed with arbitrary units. Data are representative of at least three experiments. (**e**) Thrombin generation in plasma obtained from HB mice treated without (HB) or with AAV8-SaCas9 (AT) (AT sgRNA) was assessed by cleavage of the fluorogenic substrate, and is expressed as arbitrary units. Values are mean ± SEM (n = 4). Thrombin generation in mouse plasma containing indicated concentrations of FIX:C (50%, 25%, 10%, 1%, and 0%) was assessed as a reference. **P < 0.01 (two-tailed Student’s *t*-test).

To correct the bleeding tendency of haemophilia using a single AAV vector system, we examined whether disruption of the AT gene (*Serpinc1*) by CRISPR/Cas9 could be used for haemophilia. In this series of experiments, we used haemophilia B mice, which lack a large portion of *F9* (exon 1–3) (Jackson Laboratory, Sacramento, CA, USA), for a case that cannot be treated by targeting intron 1. A synthetic small interfering RNA targeting *Serpinc1* has been suggested as an alternative drug for the treatment of haemophilia^22^. We inserted sgRNA sequences for *Serpinc1* (Extended Table 1) in the same AAV vector system and intravenously injected it into haemophilia B mice. Plasma AT activity (AT:C) clearly decreased after vector injection, and DSB in liver genomic DNA were confirmed (Fig. 4a and b). AT reduction improved fibrin formation after endothelial disruption *in vivo* and plasma thrombin generation *ex vivo* (Fig. 4c–e, and videos S1–S3). This indicates that inhibition of an anticoagulant, such as AT, using CRISPR/Cas9 could be an alternative strategy for treating haemophilia.

## Discussion

In this study, we applied a genome editing approach using CRISPR/Cas9 in an AAV8 vector and found that chromosomal DNA in hepatocytes could be efficiently modified. To treat hemophilia B mice using the CRISPR/Cas9 system, insertion of the target sequence at the DSB using NHEJ was more effective at increasing plasma FIX levels compared with HDR, even in neonates. Disruption of the *Serpinc1* gene by a single AAV vector system restored hemostasis in hemophilia B mice. These genome editing techniques have the potential to expand current AAV vector-mediated gene therapy in children with hemophilia.

The single AAV8 vector system expressing SaCas9 and sgRNA could disrupt the *F9* gene in most hepatocytes *in vivo*, reducing the levels of FIX:C to only 2–5% after administration. AAV vectors have many advantages over other vectors, including gene transferability to quiescent cells such as adult hepatocytes, no pathogenicity, and low immunogenicity^23^. Although the first study to incorporate SaCas9 into the AAV vector used the TBG promoter to transduce hepatocytes^17^, in the current study we expressed SaCas9 using the stronger HCRhAAT promoter. Recently, CRISPR/Cas9-mediated somatic correction of *F9* by hydrodynamic injection of naked DNA has been reported^15^. In that study, the researchers used hydrodynamic administration of the plasmid or oligonucleotide, which is not a realistic application in human therapy. Additionally, adenoviral expression of Cas9 did not show any therapeutic effect because of severe hepatic toxicity^15^. To solve these problems, AAV-mediated genome editing for the expression of SaCas9 has the advantage of delivering the genome editing tools to liver cells of hemophilic patients *in vivo*.

To treat genomic diseases by postnatal genome editing *in vivo*, NHEJ-related repair mechanisms using DSB should be applied. NHEJ is the main mechanism used to repair DSB^10^, and HDR rarely occurs in postmitotic adult tissues, such as skeletal muscle and the liver^3^. Although HDR mainly occurs in the S/G_2_ phase of the cell cycle^20^, HDR-dependent repair mechanisms could not sufficiently function in neonates where hepatocytes proliferate extensively. The mechanisms and molecules involved in NHEJ have been determined, and suppression of NHEJ using an inhibitor or RNA interference has been shown to facilitate HDR^24, 25^. However, expression of adenovirus E1B55k and E4orf6 proteins by AAV8 vector to increase HDR failed to increase FIX in hemophilia B mice (FIX:C: 0.578 ± 0.123; n = 4). Expression of the cDNA sequence without homology arms more efficiently improved the hemophilia phenotype, suggesting that the most straightforward way to treat genetic diseases by genome editing is to enhance the insertion of the target sequence at DSB. Recently, a homology-independent targeted integration (HITI) strategy has been developed^26^. HITI employed the insertion of a sgRNA recognition sequence into the donor sequence to enhance the integration of the target sequence^26^. Another type of RNA-guided nuclease Cpf1 creates a staggered end with a 5′ overhang, in contrast to the blunt end generated by Cas9^27^. To generate sticky ends for both donor sequences by Cpf1, targeted insertion of the sequence may be augmented.

The application of CRISPR/Cas9 overcomes the defects of current AAV vector-mediated gene therapy. AAV vectors are being widely applied to treat a range of diseases, including hemophilia, neurological diseases, cystic fibrosis, and age-related macular degeneration^28, 29^. AAV vectors are predominantly maintained episomally; therefore, cell division will dilute the AAV genome, resulting in the loss of therapeutic expression^21^. Current hemophilia gene therapy using AAV vectors is only applicable to adult patients because their hepatocytes are largely quiescent. That said, the therapeutic effects of genome editing will persist permanently, even if the vector exists only transiently within cells.

Genome editing of neonates or infants using the AAV vector may provide several important benefits. First, it would be unnecessary for such patients to receive regular replacement therapy of coagulation factor concentrates, beginning as early as 1-year-old. Second, the reduced levels of the AAV genome in the liver via cell proliferation may minimize the possibility of unwanted adverse effects by continuous expression of the nuclease. Third, there will be improved cost-effectiveness because of the reduction in the vector dose. In the current study, we administrated a 5% vector dose to newborn mice and obtained the same treatment effects. Finally, the beneficial effects of using an AAV vector is more likely in younger patients because of the lower frequency of anti-AAV neutralizing antibody^30^.

CRISPR/Cas9 has several advantages compared with zinc finger nuclease (ZFN) or transcription activator-like endonuclease (TALEN) in genetic diseases, despite sharing a similar concept to create DSB at genomic DNA. ZFN-mediated gene editing has previously been reported in a mouse model of haemophilia B^31–33^. These studies inserted a DNA sequence (cDNA of *F9*) into ZFN-mediated DSB at a safe genomic locus (artificially inserted human *F9* at *Rosa26* or *Alb*)^31–33^. Despite efficient gene editing characteristics, ZFN has not been widely adopted because of the difficulty in constructing zinc finger domains that bind a comprehensive stretch of nucleotides with high affinity^34^. To induce DSB at different sites using ZFN or TALEN, vector construction must start from the beginning. Distinct from protein-guided cleavage, CRISPR/Cas9 uses different sgRNA sequences for DNA cleavage. Because only programmable RNA is required to generate sequence specificity, CRISPR/Cas9 is easily applicable and develops quickly^35^. Indeed, we effectively induced DSB at different target sites by only changing the sgRNA sequence in the same vector system. The CRISPR/Cas9 system may be able to provide personalized genome editing to modify patient-specific gene abnormalities.

Some limitations of this study merit discussion before the clinical application of this procedure. Of particular concern with the CRISPR/Cas9 system is unwanted mutations by off-target effects. We assessed 28 potential off-target sites using the Surveyor^®^ assay and did not detect any mutations (Extended Fig. 5). We checked the sensitivity of the Surveyor^®^ assay and found that at least 5% of mutations could be detected (Extended Fig. 8). Since the possibility of marginal off-target mutations cannot be completely excluded, off-target mutations should be carefully assessed using a more sensitive and reliable assay before applying genome-editing in human therapy. Further, host immune responses triggered by Cas9 expression may attenuate the therapeutic effect or lead to uncertain adverse effects^36^. In this context, transient expression of Cas9 such as neonatal genome-editing by AAV vector may be more desirable. Finally, AAV vector may be integrated into the host genome leading to genotoxicity, albeit at a much lower frequency compared with retroviral vector^37^. Although extremely low-level AAV vector integration may be of concern, several types of AAV serotype vectors are currently being used in 173 clinical trials, and there has not been any reported increase in the incidence of cancer^38^. While we did not search for random integration of AAV after genome-editing, we did not observe any evidence of tumours during the observation period (up to 12 months). These data imply the safety of AAV vector in clinical practice. However, further studies are required to examine the safety of the expression of genome editing tools by AAV transduction in the liver.

In summary, we successfully treated hemophilic mice to deliver genome-editing tools of the CRISPR/Cas9 using AAV vector *in vivo*. The permanence of the therapeutic effect is an advantage of using CRISPR/Cas9 because it induces a modification in genomic DNA, unlike conventional AAV8 vector treatment. Further evaluation to increase the efficiency of HDR or targeted insertion and experiments using larger animal models will be necessary before this technology can be applied to hemophilia patients.

## Methods

### Animals

All animal protocols were approved by the Institutional Animal Care and Concern Committee at Jichi Medical University, and animal care was in accordance with the committee’s guidelines. C57BL/6 J mice were purchased from Japan SLC (Shizuoka, Japan). Coagulation factor IX (FIX)-deficient mice (B6.129P2-*F9* ^*tm1Dws*^) were obtained from The Jackson Laboratory (Sacramento, CA, USA).

### Design of guide RNA sequences and DNA constructs

sgRNA sequences were designed using online software provided by Thermo Fisher Scientific (Waltham, MA, USA) or Benchling (https://benchling.com) (Extended Table 1). DNAs encoding sgRNAs (trans-activating RNA-crisper RNA chimera) under the control of the U6 promoter were synthesized by GenScript (Piscataway, NJ, USA). PCR fragments of sgRNA sequences without the U6 promoter were inserted into pCR2.1 TOPO, and sgRNAs were generated *in vitro* transcription [CUGA^®^7 *in vitro* Transcription Kit (Nippon Gene, Tokyo, Japan)].

### Generation of CRISPR/Cas9-mediated gene-modified mice

A mixture of codon-optimized *Streptococcus pyogenes* Cas9 (SpCas9) mRNA (20 ng/µl; Thermo Fisher Scientific) and an sgRNA (5 ng/µl) was injected into the cytoplasm of zygotes. The zygotes were cultured until the two-cell stage, and then transferred into pseudopregnant female mice.

### Surveyor^®^ nuclease assay

Genomic mutations were detected using the Surveyor^®^ Mutation Detection Kit (Integrated DNA Technologies, Skokie, IL, USA). Briefly, PCR fragments were amplified with ExTaq DNA polymerase (Takara Bio, Otsu, Japan). Equal amounts of test and reference PCR products were denatured and re-annealed using a thermal cycler, and then treated with Surveyor^®^ Nuclease. DNA fragments were analyzed by agarose gel electrophoresis. The oligonucleotide primer sequences used to detect mutations are described in Extended Table 1.

### Plasmid constructs and AAV vector production

A cDNA of codon-optimized *Staphylococcus aureus* Cas9 (SaCas9) was kindly provided by Dr. Nureki (The University of Tokyo)^17^. A DNA fragment consisting of a chimeric promoter (HCRhAAT; an enhancer element of the hepatic control region of the Apo E/C1 gene and the human anti-trypsin promoter)^16^, SaCas9 cDNA, the SV40 polyadenylation signal, and an sgRNA sequence driven by the U6 promoter was introduced between inverted terminal repeats into the pAAV2 plasmid (See Fig. 2a). The genes were packaged by triple plasmid transfection of human embryonic kidney 293 cells to generate the AAV8 vector (helper free system), as described previously^16^. Titration of recombinant AAV vectors was carried out by quantitative PCR. The sequences of SV40 polyadenylation signal-specific primers are described in Extended Table 1.

### Targeted deep sequencing

Genomic DNA fragments that include the nuclease target sites were amplified using a KAPA HiFi™ HotStart PCR kit (KAPA Biosystems, Wilmington, MA, USA). The primer sequences are described in Table S1. PCR products were purified using AMPure^®^ XP beads (Beckman Coulter, Brea, CA, USA). A library was prepared to amplify the target region and to add Illumina sequencing adapters and dual-index barcodes to the amplicon target. PCR amplicons were subjected to 300 pair-end read sequencing using Illumina MiSeq (100,000 reads). Sixty sequences near the target sequence were extracted, and the frequency of each sequence was calculated.

### Measurement of coagulation factor activity and thrombin generation assay

Plasma FIX:C was measured with a one-stage clotting-time assay by an automated coagulation analyzer (Sysmex CA-510 analyzer; Sysmex, Kobe, Japan) using FIX-deficient plasma (Sysmex). AT:C was measured using Testzym^®^S ATIII (Sekisui Medical Co., Ltd, Tokyo, Japan). Thrombin generation was assessed as previously reported^39^. Briefly, 80 µl of mouse plasma was incubated with 20 µl of trigger reagent (5 pM recombinant human tissue factor and 40 µM phospholipid mixture) for 10 min at 37 °C. The assay was started by the addition of 20 µl 100 mM CaCl_2_ and 5 mM of the thrombin substrate Z-Gly-Gly-Arg-AMC (Wako Pure Chemicals Industries, Osaka, Japan). A fluorescent signal (excitation 390 nm and emission 460 nm) was monitored at 10 sec intervals using a Spark 10 M multimode microplate reader (Tecan, Männedorf, Switzerland).

### Histological analysis

Mice anesthetized with isoflurane were perfused with 50 ml phosphate buffered saline, and then tissues were fixed with 10% formalin. Paraffin-embedded tissue samples were sectioned and processed for hematoxylin-eosin staining or immunostaining. The SaCas9 used in this study was conjugated with hemagglutinin (HA). The sections were pretreated with 5% donkey serum, and then treated with anti-HA polyclonal antibody (MBL, Nagoya, Japan). Immunoreactivity was detected with Simple Stain Mouse MAX-PO (Nichirei Bioscience, Tokyo, Japan), and DAB (Agilent Technologies, Santa Clara, CA, USA), followed by counterstaining with Myer hematoxylin. Tissue sections were observed with an all-in-one microscope (BIOREVO BZ-9000, KEYENCE, Tokyo, Japan) at 400x magnification.

### Bleeding volume

The distal tail tip (5 mm) of an anesthetized mouse was clipped, and the tail was immediately immersed in 50 ml of phosphate buffered saline at 37 °C. Tail bleeding volumes were defined as red blood cell numbers in phosphate buffered saline for 10 minutes. The experiment was terminated by electrocauterization to prevent animal death.

### Intravital microscopy

Intravital microscopy was performed to analyze fibrin formation *in vivo*. Briefly, Rhodamine B isothiocyanate-dextran (5 mg/body; Sigma Aldrich, St. Louis, MO, USA), Hoechst 33342 (3 mg/body; Thermo Fisher Scientific), and Alexa 488-conjugated fibrinogen (300 μg/body; Thermo Fisher Scientific) were injected into anesthetized mice. Sequential images of testicular vein (at least 80 µm diameter) were obtained using a resonance scanning confocal microscope (Nikon A1RNP; Nikon, Tokyo, Japan) after local endothelial disruption induced by laser irradiation (wavelength 700 nm). The signal intensity of fibrin formation (shown by Alexa 488-conjugated fibrinogen signals) was quantified using NIS-Elements AR 3.2 (Nikon).

### Statistical analysis

Unless otherwise stated, values express mean ± SEM. Statistical analysis was by Student’s *t*-test or one-way repeated-measures ANOVA with *post hoc* Bonferroni test, as indicated in Figure legends.

## Electronic supplementary material

Video 1 Thrombus formation after endothelial disruption in wild-type mouse.

Video 2 Thrombus formation after endothelial disruption in haemophilia B mouse.

Video 3 Thrombus formation after endothelial disruption in haemophilia B mouse treated with AAV8-SaCas9 to induce DSB in antithrombin gene.

Supplementary information
