# Supplementary material for: CRISPR/Cas9-mediated genome editing via postnatal administration of AAV vector cures haemophilia B mice
Source: Sci Rep. 2017 Jun 23;7:4159. doi: 10.1038/s41598-017-04625-5 (PMC5482879; doi:10.1038/s41598-017-04625-5)
Supplement: Supplementary file 4 — Supplementary information [file 41598_2017_4625_MOESM4_ESM.pdf]

CRISPR/Cas9-mediated genome editing via postnatal administration of AAV vector cures haemophilia B mice

Tsukasa Ohmori[1,*], Yasumitsu Nagao[2], Hiroaki Mizukami[3], Asuka Sakata[4], Shin-ichi Muramatsu[5,6], Keiya Ozawa[6], Shin-ichi Tominaga[1], Yutaka Hanazono[7], Satoshi Nishimura[4,8,9], Osamu Nureki[10], Yoichi Sakata[4]

[1] Department of Biochemistry, Jichi Medical University School of Medicine, Tochigi, 329-0498, Japan

[2] Center for Experimental Medicine, Jichi Medical University, Tochigi, 329-0498, Japan

[3] Division of Genetic Therapeutics, Center for Molecular Medicine, Jichi Medical University, Tochigi, 329-0498, Japan

[4] Division of Cell and Molecular Medicine, Center for Molecular Medicine, Jichi Medical University, Tochigi, 329-0498, Japan

[5] Department of Neurology, Jichi Medical University School of Medicine, Tochigi, 329-0498, Japan

[6] The Institute of Medical Science, The University of Tokyo, Tokyo, 108-0071, Japan

[7] Division of Regenerative Medicine, Center for Molecular Medicine, Jichi Medical University, Tochigi, 329-0498, Japan

[8] Department of Cardiovascular Medicine, The University of Tokyo, Tokyo, 113-8655, Japan

[9] Translational Systems Biology and Medicine Initiative, The University of Tokyo, Tokyo, 113-8655, Japan

[10] Department of Biological Sciences, Graduate School of Science, The University of Tokyo, Tokyo 113-0032, Japan

Supplemental Materials

Extended Table 1-3

Extended Figure 1-8

Video 1-3

Extended Table 1 sgRNA and the oligonucleotide primer pairs used in this study.

Taget gene	Cas9	sgRNA	Target site	Sequence	PAM sequence
F9	*Streptococcus pyogenes*		Exon 8	GGTTTCCCGGTACGTCAAC	TGG
F9	*Staphylococcus aureus*	sgRNA-1	Exon 8	TCAACAAAGGGAGACAGGCTT	CCATTC
		sgRNA-2	Exon 8	CAGTACCTTAGAGTTCCACTG	GTGGAT
		sgRNA-3	Exon 8	TAAGGTTTCCCGGTACGTCAA	CTGGAT
F9	*Staphylococcus aureus*	sgRNA-1	Intron 1	TTGATCCCGAGGGTCTATACA	GTGAAT
		sgRNA-2	Intron 1	CAGGAGACCAGCCGATTTTCT	GGGGAT
		sgRNA-3	Intron 1	TCCCTCACCACTAAGACGTGC	TTGGAT
Serpinc1 *(antithrombin)*	*Staphylococcus aureus*	sgRNA-1	Exon 8	GAGGAAGGCAGTGAAGCAGCA	GCGAGT

Target DNA		Sequence
Surveyor Assay		
Exon 8 of mouse *F9*	F	5'- AACTGGGCAAATGGGAGAG -3'
	R	5'- TCAGGAGAGGAAGTCATGC -3'
Intron 1 of mouse *F9*	F	5'- AACAGTGGCATTACTCCCCA -3
	R	5'- CCAAACTGGCCTGTGAGAAA -3
Exon 8 of mouse *Serpinc1*	F	5'- GTGGTAGTGATAGCTGGGAT -3'
	R	5'- GGGAGATTGATTCGGGTTTG -3'
Exon 3 of mouse *Serpinc1*	F	5'- CCTTGTCTGGGGTTTTCTGA -3'
	R	5'- GATCACTGGGTGTCTTTCCA -3'
Real time qPCR		
SV40 polyA	F	5'- AGCAATAGCATCACAAATTTCACAA -3'
	R	5'- CCAGACATGATAAGATACATTGATGAGTT -3'
	probe	5'- AGCATTTTTTTCACTGCATTCTAGTTGTGGTTTGTC -3'
Deep sequencing		
Exon 8 of mouse *F9*	F	5'- TCGTCGGCAGCGTCAGATGTGTATAAGAGACAGTGATCAGTGAAGCCAACCAGACTGGG -3'*
	R	5'- GTCTCGTGGGCTCGGAGATGTGTATAAGAGACAGCCTTCACACGAATCTTTGCCTCCTTC -3'*
Exon 8 of mouse *F9* (HDR frequency)	F	5'-TCGTCGGCAGCGTCAGATGTGTATAAGAGACAGNNNNNNNNGTAACACCTATCTGTGTTGCCAATAGGG-3'**
	R	5'- GTCTCGTGGGCTCGGAGATGTGTATAAGAGACAGCTGTAAAGGGCATCACCCATTTTCAAT -3'
Detection of HDR and NHEJ		
	F	5'- GGGATCTACACCAAAGTGAG -3'
	R	5'- CCAAACTGGCCTGTGAGAAA -3
Detection of coF9 mRNA		
	F	5'- ATGAAGCACCTGAACACCGT -3'
	R	5'- CCAGTTCACGTATCTGCTCA -3'

*Overhang adapter sequences were appended to the primer pair sequence (underline). ** NNNNNNNN means barcode sequence. coF9, codon-optimized F9

Extended Table 2 Frequency of *F9* genomic sequences in liver with the administration of AAV vector encoding SaCas9 and sgRNA for *F9* (Exon 8).

Genomic DNA sequence	sgRNA2 (%)
AAGGGAGACAGGCTTCCATTCTTCAGTACCTTAGAGTTCCACTG**GTGGAT**AGAGCCACAT	33.4
AAGGGAGACAGGCTTCCATTCTTCAGTACCTTAGAGTTCCA--G**GTGGAT**AGAGCCACAT	19.8
AAGGGAGACAGGCTTCCATTCTTCAGTACCTTAGAGTTCCA-TG**GTGGAT**AGAGCCACAT	6.38
AAGGGAGACAGGCTTCCATTCTTCAGTACCTTAGAGTTCCAACTG**GTGGAT**AGAGCCACAT	5.06
AAGGGAGACAGGCTTCCATTCTTCAGTACCTTAGAGTTCCA---**GTGGAT**AGAGCCACAT	3.33
AAGGGAGACAGGCTTCCATTCTTCAGTACCTTAGAGTTC--CTG**GTGGAT**AGAGCCACAT	2.29
AAGGGAGACAGGCTTCCATTCTTCAGTACCTTAGAGTT---CTG**GTGGAT**AGAGCCACAT	1.52
AAGGGAGACAGGCTTCCATTCTTCAGTACCTTAGAGTTCCATCTG**GTGGAT**AGAGCCACAT	1.36
AAGGGAGACAGGCTTCCATTCTTCAGTACCTTAGAGTTCCA---**-TGGAT**AGAGCCACAT	1.13
AAGGGAGACAGGCTTCCATTCTTCAGTACCTTAGAGTTCCA---**---GAT**AGAGCCACAT	1.09

* Table shows sequences more than 1%. Underline and bold mean sgRNA and PAM sequence, respectively.

Extended Table 3. The oligonucleotide primer pairs used to detect off-target sites.

No	Potential target site*	Chr	Position	Direct	Mismatches	Bulge Size	F primer	R primer	Product Size (bp)
1	aAGTAgCTTAGAGTTCCACatCTGGAT	chr8	23559019	+	4	0	CGGCCATACTCAGAGACACT	ATTTCCCAAGACCCCTTCCA	584
2	gAGTcaCTTAGcGTTCCACTGTAGAGT	chr3	145842543	+	4	0	CCACTAACCCAGGCAAGAGA	GGCTGCCTACTGAGAACTGA	485
3	CAGTtCCTTAcAtTTCCACaGGAGGGT	chr7	133596743	−	4	0	TCCCCTCTCCCTGTTTTGTG	CCTGTGACCCTCCTCCTTTC	537
4	CAGTcCaTTAcAGgTCCACTGGAGAGT	chr4	20497305	−	4	0	CTCTTCAGAAACTGCAGGAAGT	GAGTTCTTGCTGCAAAATTCCC	465
5	CAGgACCTTAGAGgTCaAaTGGTGGAT	chr4	62455062	−	4	0	TCAGGACCTTCAGAAGAGCA	AGGGGAACTGAGTTTCTGGAG	535
6	CAGTcCCTTAGgaTgCCACTGCTGGAT	chr4	88325172	−	4	0	TGCAACACATTTTCCCAGGG	TCCTTCTCAGCGGGATTTGT	484
7	aAGTAgCTTAGAGTTCCACacCTGGAT	chr4	93806662	+	4	0	CAAGAAGTAGGAGCGGGTGG	GGCAAATGGATGTATCTGGAGG	584
8	aAGTACCTaAGAGTTCCACatCTGGAT	chr5	73661304	+	4	0	CAACCCCAAGTGGTGCTG	CCCAGTTGCTAGCCCTACTA	525
9	CAGTACCTTAGAGTatCtCTcAGGGGT	chr16	23623100	−	4	0	TTAGAATTGGGGCCTTTGGG	CAGTTGGGGCATCTTCTGGA	517
10	CAGTACCTaAcAGTTCCAtcGTGGAAT	chr1	41489284	+	4	0	AGGGGTAGTTCAGCTTCACT	TGTCTGAGAGTGGGCATGAA	468
11	aAGTAgCTgAGAGTTCtACTGAAGAGT	chr10	105187353	−	4	0	TATGTTGTGTGGGGCCTTCT	CGGGACTCTATAGCATCCTGT	483
12	CAGgACCcTAGAGTgCCACaGCTGAAT	chr14	28134496	−	4	0	ATCTCTGCCCTGGTGTTCAT	GTGGCTAGAGGTCAGCAGAA	478
13	CAGTtCCccAGAGTTCCACTGTGGAGT	chr14	79201827	+	3	0	GCAAACCCTCCCAACATGTT	CATGTCAGAGCAGCGTTTCA	534
14	CAGTACCTcAGAGaTCtACTcCAGAGT	chr18	83818780	−	4	0	CTGACTGGGAAAAGGAGGAGT	AACGTGCAGCTAAAGGACAC	578
15	CAGTAgCTTAGAGTTCtACatCTGGAT	chr11	58405590	+	4	0	CACAGAACACGTGCTGAACA	CCCCATAGGCTCACAGATTTG	453
16	CAGTAgCaTAGgGTgCCACTGGGGGGT	chr11	78154002	−	4	0	CATGCAGCAAGGGATTAGGG	AACTGCTCACTATCCCACCC	581
17	CAGaAgCTT--AGTTCCACTGTAGAGT	chr12	67297545	+	2	2	CAACCTGAGTGCTGCTGTTG	AGTGGGAGGGAAGGAAGACT	579
18	CAGTACCTTAGA-TaCCACaGTTGAGT	chr1	121171721	−	2	1	AACCTGGCTGTACGTGACTT	AGAGTGAAGTCTGGTGGCTT	592
19	CAGgACCTTAGtGT--CACTGGTGAGT	chr2	14513072	−	2	2	ATTGGGGCAGTGGTCTCTAC	GGTTGGGCAGGTTTCTTGAG	596
20	CAG--CCTTAGAGTTCtAtTGGAGAAT	chr2	102645587	−	2	2	TCAGGAGCAAAGGTGATGGT	GGTGACACCCATCAACGTAT	477
21	CAG--CCTcAGAGTTCCAgTGTGGGAT	chr15	97324417	+	2	2	GATGTCCTTCTCTTCGCACAC	ATGTCCTTCCCTCCCCAATC	600
22	CAGTACtTTAGAtTTC--CTGTTGAAT	chr6	64406534	+	2	2	ACCTTGGTGTGTTTGCTGTT	AGTTAGCCTGGGGACTTTTC	503
23	CAGTtCCT--GAGTTCCAgTGCAGAGT	chr9	7855517	−	2	2	TTAACTGACAGGGCAGAGCA	TGTTTGTGAGGGTCAAGGAC	457
24	CAGgACCTTAGgGT--CACTGGTGAGT	chr9	4276120	+	2	2	AGCTTATCCAGGAACCACTCT	GGCTCCTGTGAACCTGAGAT	495
25	CtGTACCTaAGAGTT--ACTGCTGGAT	chr9	102558616	−	2	2	GGATCTTGGGAGCGTCTTTC	AGGCATGACAGATTCCTCCT	496
26	CAGTAtCTTAGAaTTC--CTGGTGAGT	chr9	12553933	+	2	2	GCCAGAGTTCCAGGAGCATA	GGTGTGTCAGAGTTCTTGGC	522
27	CAcTA--TTAGAGTTaCACTGTTGAGT	chr18	52471267	−	2	2	GAAAAGTGTACACCAAAAGCGT	ATGTGCTCATCCTTGGACCA	416
28	CAGTAtCTTAGAaT--CACTGGTGAGT	chr11	13722136	−	2	2	TGTGTGTGTGTATCAGAGCCT	CTGAGACCCTGAGACCTTGG	470

*Mismatch sequence is described in small letter, and bulge is showed by a hyphen.

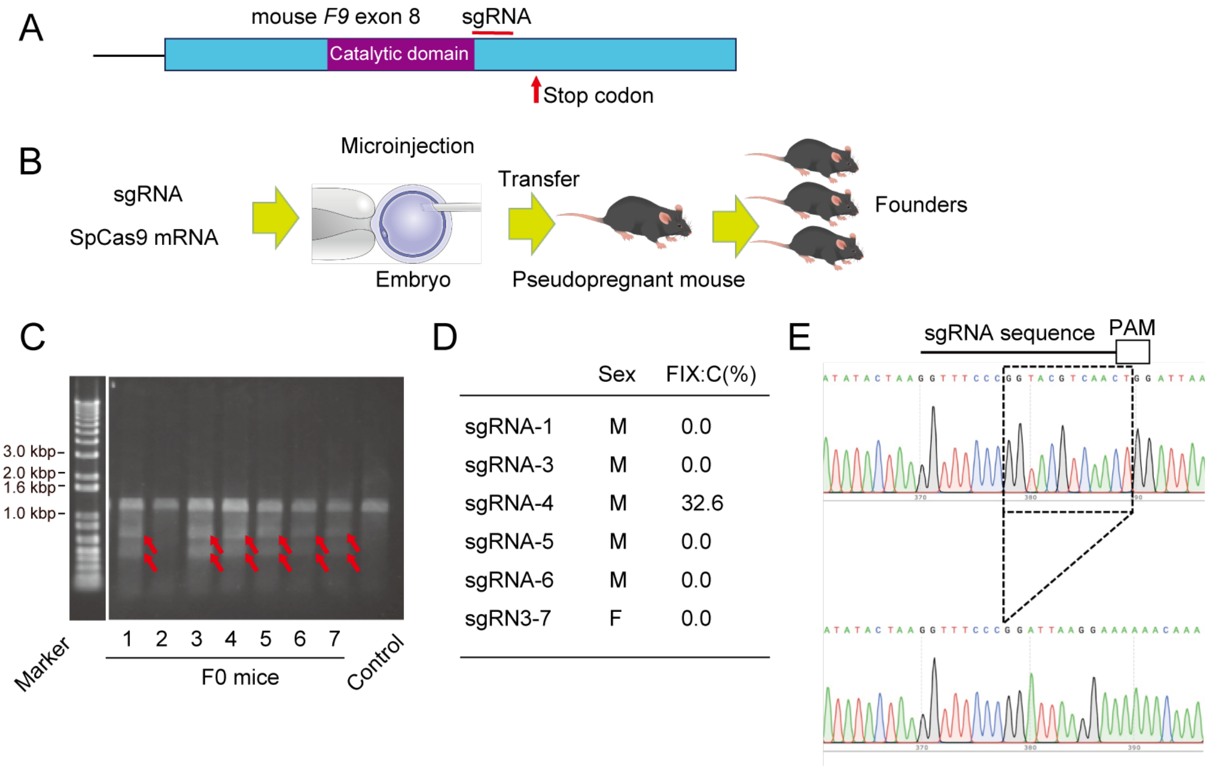

Extended Fig. 1. Generation of haemophilia B mice by injection of sgRNA and SpCas9 mRNA into zygotes

(A) Schematic diagram of sgRNA targeting exon 8 of mouse *F9*. (B) Method to generate CRSPR/Cas9-mediated haemophilia B mice. sgRNA and SpCas9 mRNA were injected into zygotes and transferred into pseudo-pregnant female mice. (C) Cas9-mediated cleavage of *F9* in founder mice detected using the Surveyor® nuclease assay. Red arrows represent a mutation. (D) Plasma levels of FIX:C in founder mice positive for the Surveyor® nuclease assay. (E) Sequence of the *F9* locus in F2 male mouse derived from a founder.

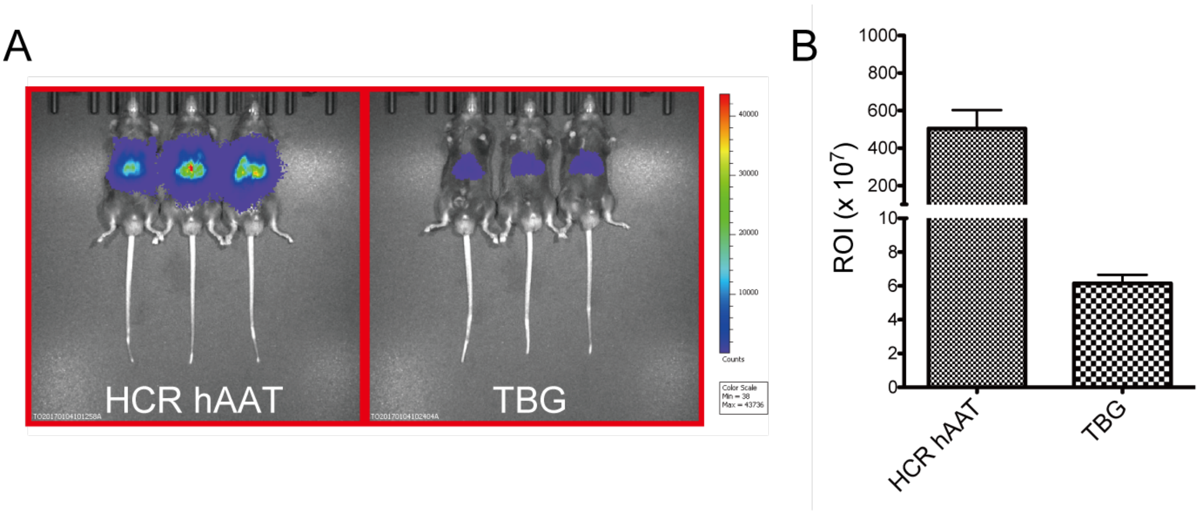

Extended Fig. 2. Comparison of promoter activity between HCRhAAT promoter and TBG promoter

AAV8 vector expressing luciferase under control of a chimeric promoter (HCRhAAT; an enhancer element of the hepatic control region of the Apo E/C1 gene and the human anti-trypsin promoter) or thyroxine-binding globulin (TBG) promoter was intravenously injected into 7-week-old C57BL/6J male mice (1×10^{11} vector genome/body). (A) *In vivo* bioluminescence images were obtained using an IVIS Imaging System at 14 days after administration. (B) *In vivo* bioluminescence of mice was quantified (photons/s). Values are mean ± SEM (n=3).

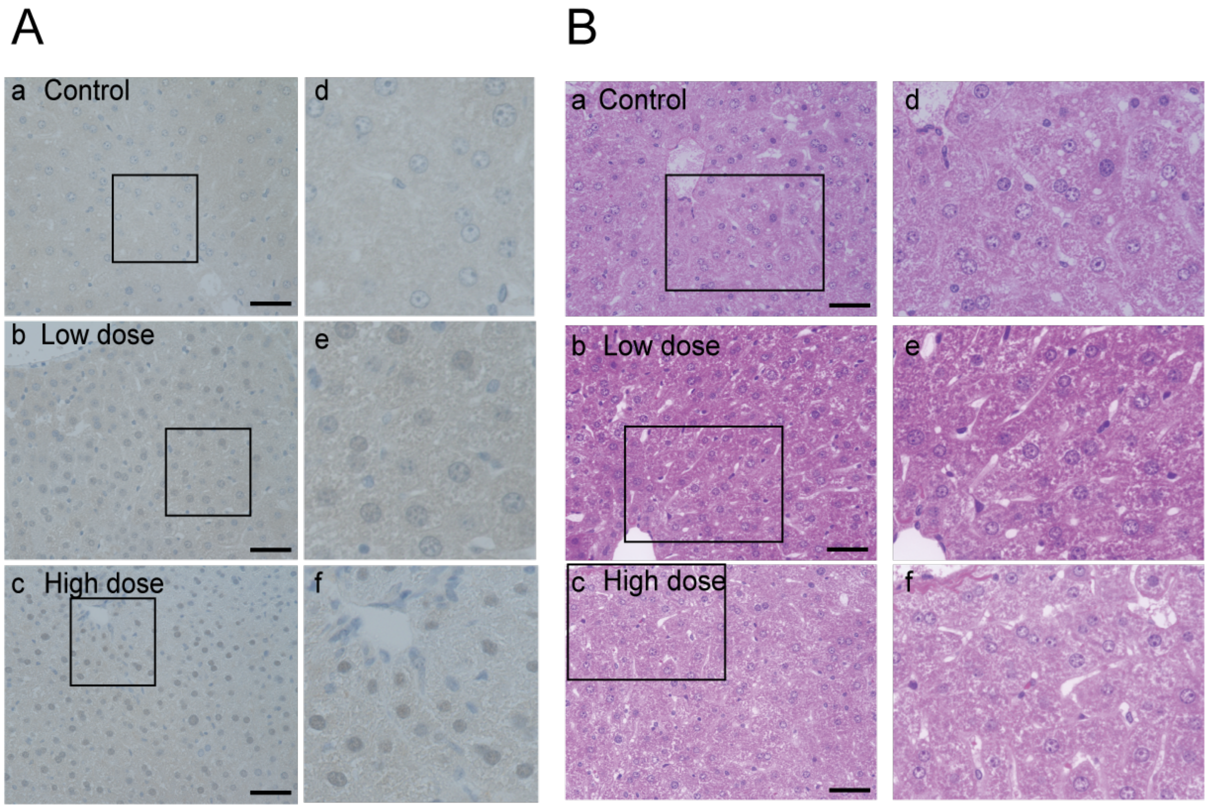

Extended Fig. 3. Expression of SaCas9 in hepatocytes and histological analysis of the liver from mice treated with AAV vector expressing SaCas9

AAV vector expressing SaCas9 and sgRNA targeting *F9* was intravenously injected into C57BL/6J mice. (A) SaCas9 expression in the liver was assessed by immunohistochemical analysis at 12 weeks after vector injection. (B) Liver sections at 12 weeks after vector injection were stained with haematoxylin and eosin. Sections were observed with an all-in-one microscope (BIOREVO BZ-9000; KEYENCE, Tokyo, Japan) at ×400 magnification. Higher magnifications of the boxed regions are shown in right-hand images. Scale bars, 50 µm. Control (a and d): C57BL/6J mouse without AAV administration; Low dose (b and e): C57BL/6J mouse treated with 3×10^{11} AAV vector genome/body; High dose (c and f): 1×10^{12} AAV vector genome/body.

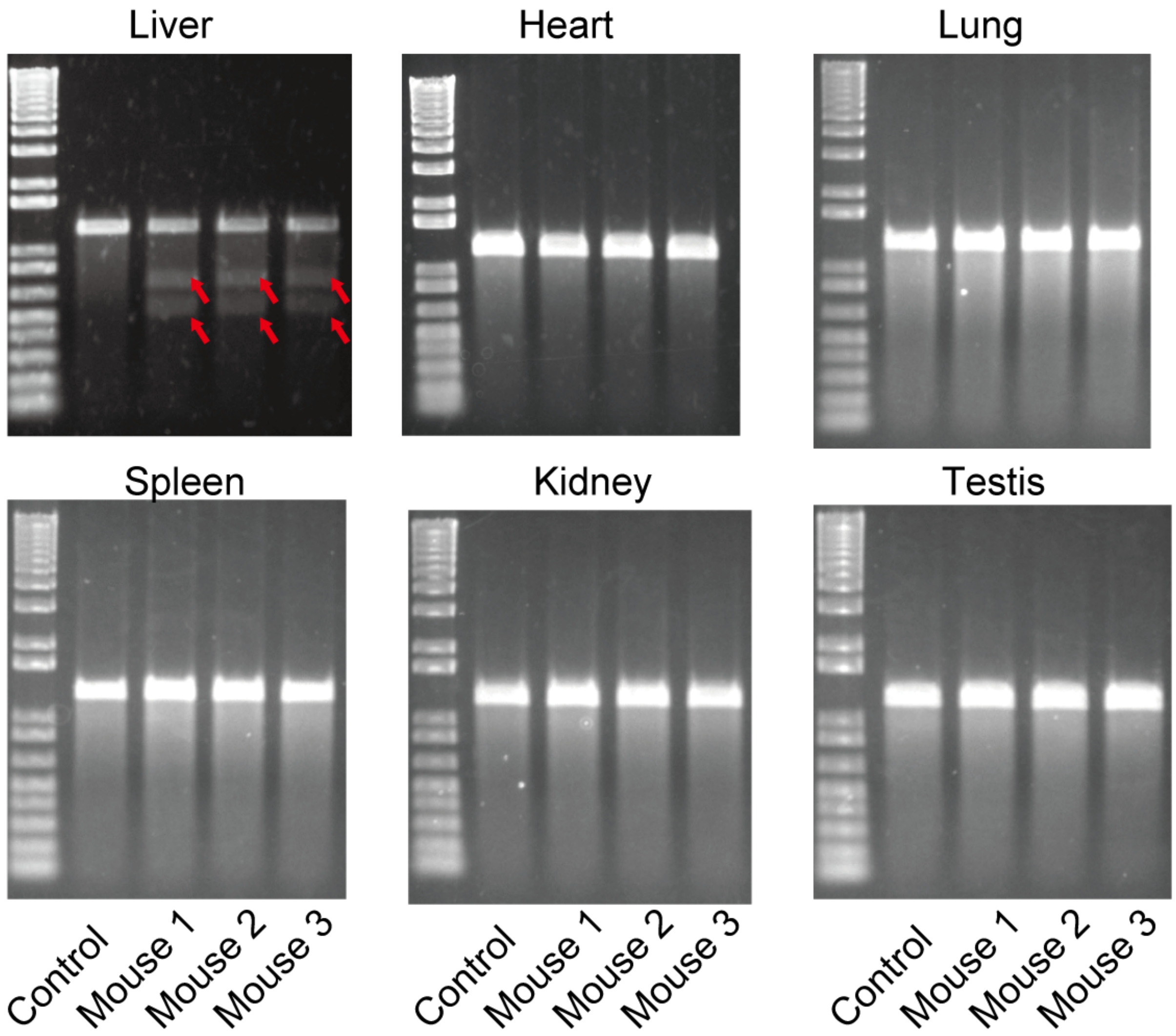

Extended Fig. 4. Liver-specific genome editing using the AAV8 vector

AAV8 vector expressing SaCas9 and sgRNA2 targeting *F9* was intravenously injected into C57BL/6J mice (1 × 10^{12} vector genome/body). Cas9-mediated cleavage of *F9* in indicated organs was assessed using the Surveyor® nuclease assay at 12–16 weeks after vector injection. Control was DNA from non-treated C57BL/6J mice. Red arrows represent a mutation.

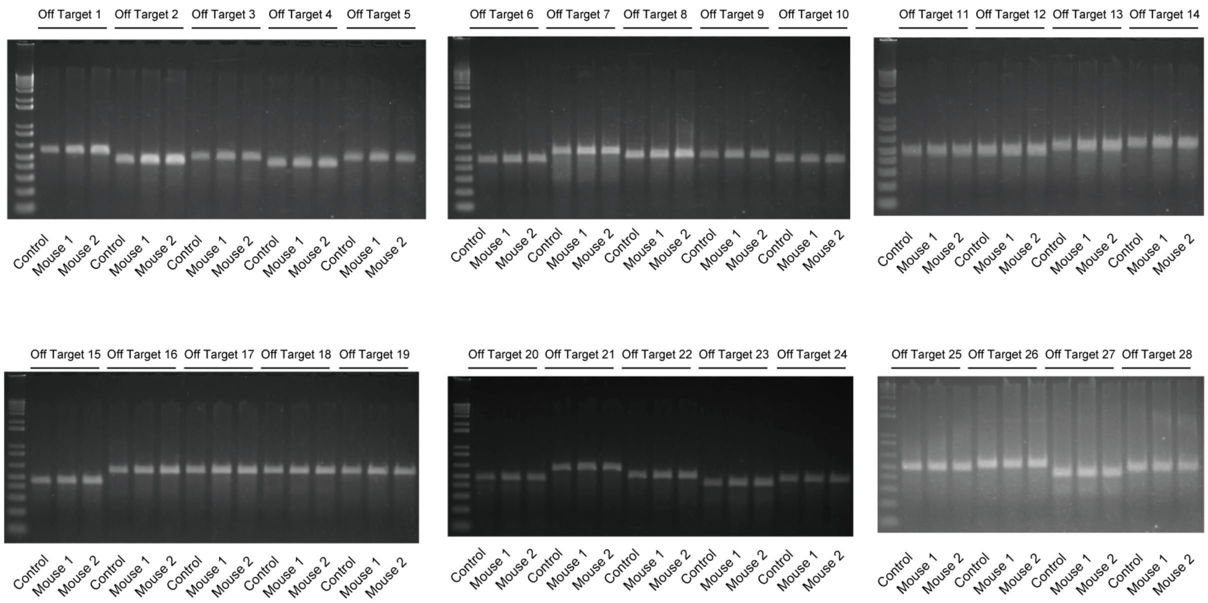

Extended Fig. 5. Surveyor® assay of potential SaCas9 off-target sites

AAV8 vector expressing SaCas9 and sgRNA2 targeting *F9* was intravenously injected into C57BL/6J mice (1×10^{12} vector genome/body). Cas9-mediated cleavage of 28 potential off-target sites was assessed using the Surveyor® nuclease assay. The same liver genomic DNA confirming non-homologous end joining were assessed (Mouse 1 and 2). Control was liver DNA from non-treated C57BL/6J mice.

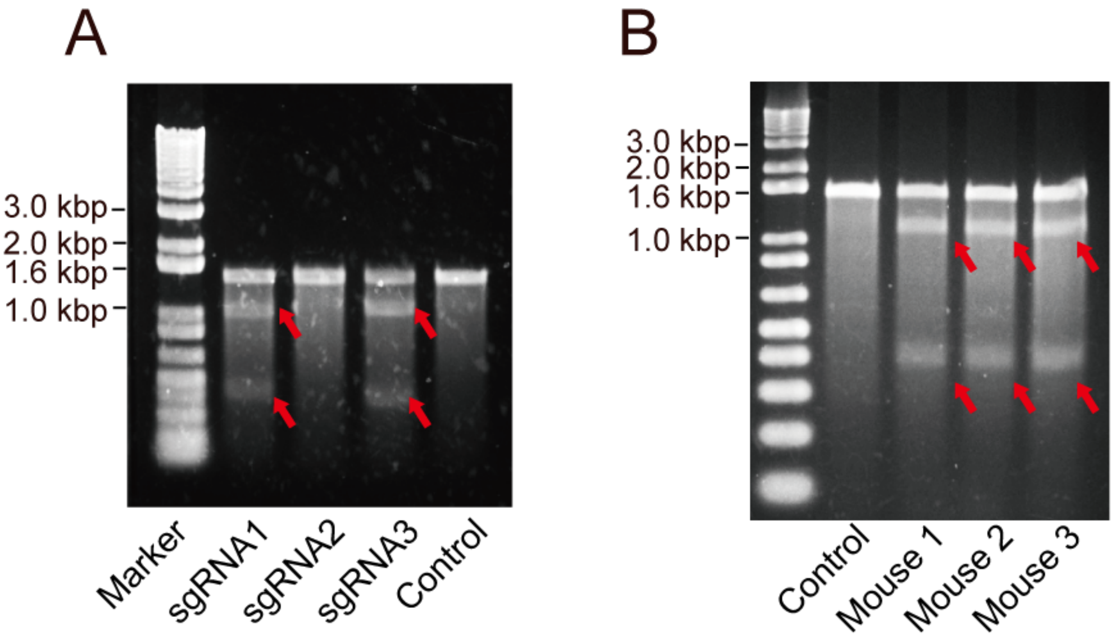

Extended Fig. 6. Determination of sgRNA sequences targeting *F9* intron 1

(A) NIH-3T3 cells were transduced with plasmid vector expressing SaCas9 driven by cytomegalovirus promoter and each sgRNA targeting *F9* intron 1. Cas9-mediated cleavage of F9 was assessed using the Surveyor® nuclease assay. (B) AAV8 vector expressing SaCas9 and sgRNA3 targeting *F9* intron was intravenously injected into 7-week-old C57BL/6J male mice and Cas9-mediated cleavage of *F9* in the liver was assessed using the Surveyor® nuclease assay. Control was liver DNA from non-treated C57BL/6J mice. Red arrows represent a mutation.

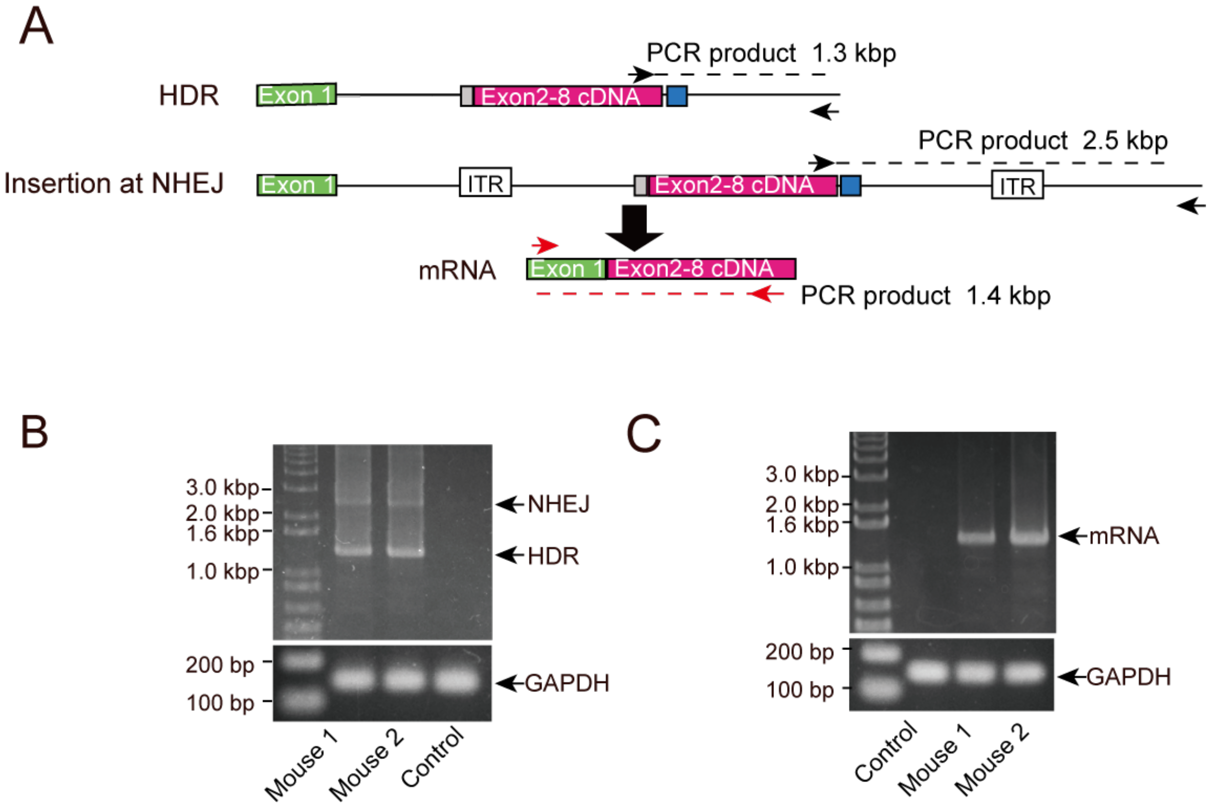

Extended Fig. 7. Genotyping of *F9* locus with HDR and insertion of template at DSB

(A) The *F9* locus was targeted by HDR and the direct insertion of template by creating a DSB in *F9* intron 1 via SaCas9 expression and supplying an AAV8 donor template. (B, C) Haemophilia B mice treated without (Control) or with AAV8-SaCas9 (intron 1) and AAV8-Targeting (Mouse 1 and 2). (B) PCR analysis of liver genomic DNA to examine HDR and insertion at DSB at 6 weeks after vector injection. Genotyping using primers (black small arrows) can distinguish HDR and the insertion by-product size. (C) RT-PCR of liver RNA to confirm expression of codon-optimized *F9* mRNA from the targeted genome sequences.

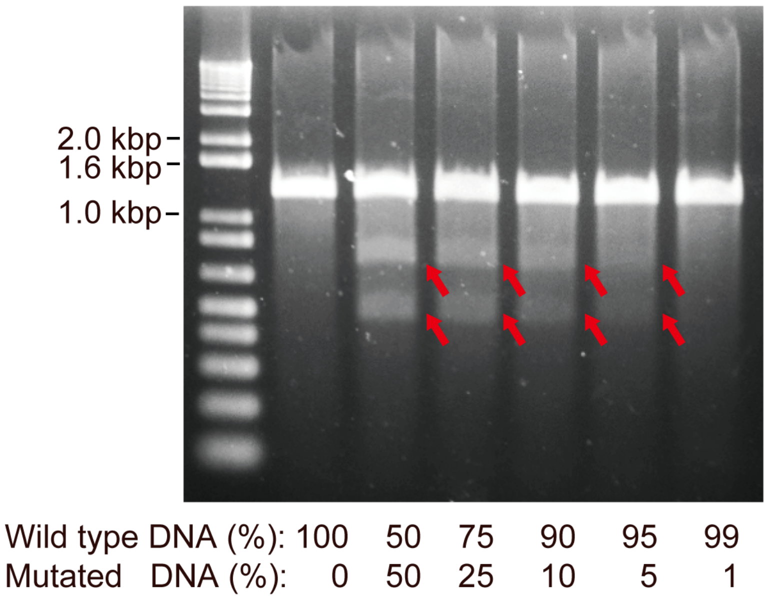

Extended Fig. 8. Sensitivity of the Surveyor® nuclease assay to detect the mutation

PCR products of *F9* liver DNA obtained from C57BL/6 mice were mixed with those of haemophilia B with the mutation at the indicated ratio. The mixed samples were denatured and re-annealed using a thermal cycler, and then treated with Surveyor® nuclease. DNA fragments were analysed using agarose gel electrophoresis. The assay could detect 5% of mutations.
